# Monitoring Menstrual Health in Footballers: Considerations for Tracking Menstrual and Hormonal Contraceptive Cycles in the Field to Support Performance

**DOI:** 10.1007/s40279-025-02338-8

**Published:** 2025-11-24

**Authors:** Ritva S. Mikkonen, Johanna K. Ihalainen, Georgie Bruinvels, Nonhlanhla S. Mkumbuzi, Abbie E. Smith-Ryan, Mira Kaikkonen, Malita Kasongo, Anthony C. Hackney

**Affiliations:** 1 Faculty of Sport and Health Sciences, University of Jyväskylä, Jyväskylä, Finland; 2 Finnish Institute of High Performance Sport KIHU, Jyväskylä, Finland; 3 Department of Targeted Intervention, University College London, London, UK; 4 Department of Sports, Exercise, and Rehabilitation, Northumbria University, Newcastle Upon Tyne, UK; 5 Department of Human Movement Science, Nelson Mandela University, Qheberha, South Africa; 6 Department of Rehabilitation, Midlands State University, Gweru, Zimbabwe; 7 NtombiSport (PTY) Ltd, Cape Town, South Africa; 8 Department of Exercise and Sport Science, Department of Nutrition, University of North Carolina, Chapel Hill, NC USA; 9 Jyväskylä Sport Academy, Jyväskylä, Finland; 10 Department of Obstetrics and Gynaecology, Mainasoko Medical Centre, Lusaka, Zambia

## Abstract

Monitoring menstrual health has gained popularity in sports like football as an opportunity to identify recurring symptoms or adverse symptoms related to the menstrual or hormonal contraceptive cycle; to recognize challenges related to low energy availability (LEA), low carbohydrate availability, overreaching/overtraining, and/or overall lifestyle stress due to their association with menstrual disturbance/dysfunction; to be informative in contextualizing athlete training status, e.g., training load and performance progression; and to promote and empower body/health literacy and overall health in female athletes. Monitoring menstrual health may also offer valuable insights to inform decisions regarding training and recovery. In team sports like football, where training loads and match schedules are relatively uniform across the squad, individualized strategies to effectively manage recurring adverse symptoms or menstrual disturbance/dysfunction may be necessary to ensure that all athletes can perform and recover optimally. The purpose of this article is to describe the rationale and suggested approaches for tracking menstrual and hormonal contraceptive cycles (including menstrual disturbance/dysfunction) in field settings to facilitate monitoring of menstrual health to potentially contextualize the other health and performance data. Herein, we assess the feasibility and potential limitations of different tracking methods from traditional paper and pencil records to more sophisticated digital applications and biochemical measures for use in real-world settings.

## Key Points

**Table:** 

Monitoring menstrual health in football and other team sports may generate meaningful data regarding the overall health and well-being of athletes. These data may be used to inform training/recovery practices in naturally menstruating and eumenorrheic athletes as well as in those experiencing menstrual disturbance/dysfunction or those using hormonal contraceptives
Monitoring menstrual health should be specific to the players’ and team’s goals and purposes. Education and rationale should be provided to players and coaching team staff in addition to multidisciplinary healthcare teams before asking for voluntary informed consent for tracking methods to be used. It must be understood that consent can be withdrawn and should have no impact on a player’s status within the team or contracts, etc
Menstrual and hormonal contraceptive tracking tools are increasingly accessible and feasible for use across diverse sports settings. However, their benefits and limitations must be clearly understood while potential cultural, societal, legal (e.g., data privacy), ethical, and financial implications should be carefully considered along with their potential to overburden players
When feasible, teams should have dedicated personnel (support staff, sports scientists) who are trained to analyze data and act ethically while contextualizing collected data for use by coaches and players. Data collection (e.g., blood samples and transvaginal ultrasound) should only be performed by qualified medical professionals or the players themselves (e.g., luteinizing hormone surge, cervical mucus, symptoms)

## Introduction: Menstrual Health and Sport

Monitoring health and performance variables in athletes is important for examining changes in adaptive responses and, when used effectively, may potentially aid in reducing the risk of illness, injury, and overtraining [1]. Tracking menstrual and hormonal contraceptive cycle characteristics (menstrual health) can be a critical aspect of overall health and performance monitoring because tracking can (1) facilitate the identification of possible patterns in adverse symptoms or side effects that can then be anticipated and possibly mitigated; (2) help to identify and address challenges related to low energy availability (LEA), low carbohydrate availability, and overreaching/overtraining as well as overall lifestyle stress due to their association with menstrual disturbance/dysfunction; (3) be informative in contextualizing athlete training status, e.g., training load and performance progression; and (4) promote and empower body/health literacy and health in female athletes, i.e., “the ability to [read], understand, and use health information to make appropriate healthcare [and training] decisions and follow instructions for treatment” [2]. That is, monitoring menstrual health can provide information to indicate if (1) an athlete needs to seek attention from their coach or a multidisciplinary healthcare team (MDT), which may consist of medical doctors/trained physicians (hereafter, physicians), nurses, physical therapists, nutritionists, etc. or (2) if a physician might need to intervene in player care. Overall, informed and properly implemented collection and interpretation of menstrual health data can help facilitate the development of athlete-centered approaches to coaching and healthcare.

For menstrual health tracking methods to be effectively implemented and benefited from, athletes, coaching staff, and MDTs must be educated regarding the menstrual cycle, menstrual disturbance/dysfunction, and hormonal contraceptive use and what implications these may have for overall health and/or performance. Education about (1) voluntary informed consent and rights related to withdrawal of consent; (2) how to potentially collect personal menstrual data [especially luteinizing hormone (LH)-surge (ovulation) kits and assessment of cervical mucus]; (3) who it is appropriate to share data with; and (4) with whom to address any possible issues related to their menstrual or overall health is essential. Communication about these issues within and around teams is necessary to avoid personal boundary transgression as well as unfounded practices such as excluding athletes from teams or training sessions at particular times across the menstrual or hormonal contraceptive cycle or ignoring menstrual disturbance/dysfunction due to outdated and false perceptions of menstrual disturbance/dysfunction being an unavoidable or even desired outcome of training [3–5]. That is, athletes, coaching staff, and MDTs must be provided with evidence-based information regarding the potential effects of (1) menstrual or hormonal contraceptive cycles on objective and subjective measures of performance; (2) menstrual cycle or hormonal contraceptive phase on training responses and adaptations; and (3) menstrual disturbance/dysfunction on health, performance, and training responses and adaptations. This information can be contextualized with information about individual subjective experiences of menstrual and hormonal contraceptive cycles as well as menstrual disturbance/dysfunction. Where possible, organizations should have dedicated resources and/or appropriate referral processes (to, e.g., physicians, including gynecologists) to avoid making menstrual health monitoring a vulnerability or liability; however, it should be recognized that the often poorly resourced nature of women's sports may mean that coaching staff and MDTs are already overstretched. Therefore, when planning monitoring and introducing tracking tools, the potential benefits and risks should be considered alongside the feasibility for each individual athlete and/or team. In all cases, it must be understood that only physicians are qualified to make medical diagnoses and that menstrual health data are medical data and should be treated as such. For more information on data handling, see Sect. 4*.*

The purpose of this article is to describe the rationale and approaches for tracking menstrual and hormonal contraceptive cycles (including menstrual disturbance/dysfunction) while also assessing the feasibility and potential limitations of different tools for use in real-world settings such as in football (soccer) teams. Providing practical application is one of our key goals; therefore, we have focused on tracking in field conditions, with only brief references to tracking for laboratory research purposes (for other methodological considerations please, see, for example, [6–8]). We provide several examples of how and what to track as well as brief practical information regarding the contextualization of tracked data. We remind the reader that interpretation of menstrual health data for diagnostic purposes should only be done by a physician. We provide a nuanced and pragmatic approach recognizing that menstrual disturbances/dysfunction and hormonal contraceptive use are relatively common in footballers as well as the general athletic population—these populations can also benefit from gathering and contextualizing data related to their menstrual health. The reader should understand that monitoring fitness/performance/recovery as well as other general health measures (e.g., hematological measures) may be recommended in athletic and physically active populations; however, the current paper will focus on references specific to menstrual health and football.

*Nota bene* In this paper, we use the terms “women” and/or “female” in line with the literature presented. We recognize that reaching populations for whom menstrual/reproductive health is essential but who do not identify as female may be challenging. As such, we encourage readers to remember that menstruation, the experience of menstrual disturbances/dysfunction, and/or unique hormone profiles that influence health and performance also exist in transgender and gender-diverse individuals.

## Menstrual and Hormonal Contraceptive Cycles

The hypothalamic-pituitary-ovarian (HPO) axis is a complex hormonal system that regulates female reproductive function via the menstrual cycle. The HPO axis includes the hypothalamus, pituitary gland, and ovaries where (1) the hypothalamus releases gonadotropin-releasing hormone (GnRH) to stimulate the pituitary gland; (2) the pituitary gland secretes follicle-stimulating hormone (FSH) and LH to stimulate the ovaries; and (3) the stimulated ovaries produce estrogens [primarily estradiol (E2) and progesterone (P4)] while orchestrating follicle development and ovulation, both of which are necessary for fertility. E2 and P4 play significant roles in regulating the endometrium (uterine lining). E2 promotes the thickening of the endometrium for potential implantation of a fertilized egg, while P4 helps stabilize and maintain the thickened lining. A decrease in these hormones at the end of the menstrual cycle causes shedding of the uterine lining known as uterine bleeding (menstruation, menstrual bleeding, menses). HPO-axis function and uterine bleeding can be regulated to a certain extent by using hormonal contraceptives, which partially or totally suppress ovarian function via exogenous hormones. The following sections will describe common sex hormone profiles in female athletes, including a brief description of how these profiles might influence athlete health and performance. While we recognize that pregnancy, lactation, puberty, the menopausal transition, and various illnesses can markedly influence hormone profiles and may be present in the athlete population, thorough discussion of these topics goes beyond the scope of this article.

### Natural or Eumenorrheic Menstrual Cycle

**Fig. 1:**
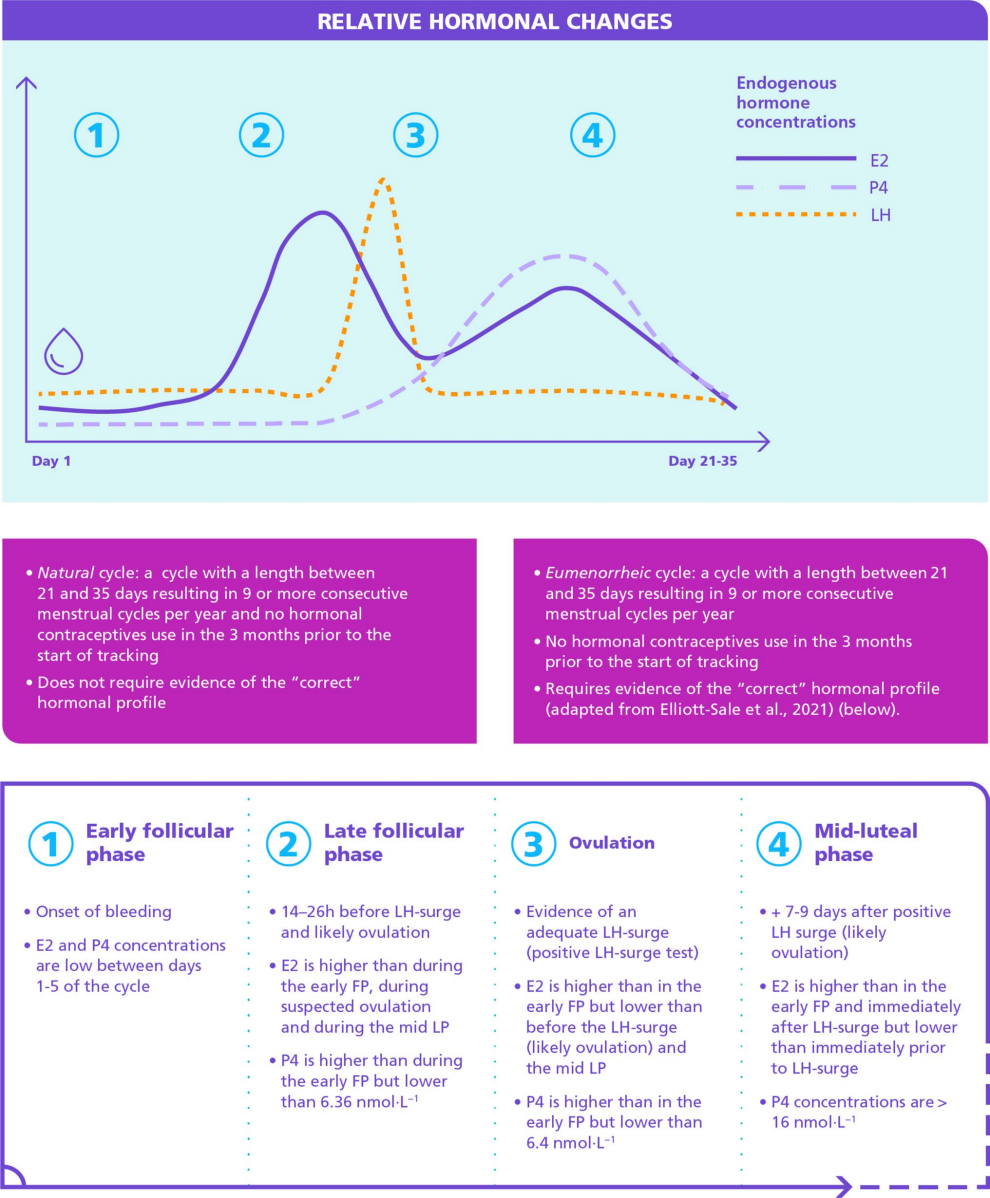
Natural or eumenorrheic menstrual cycle, adapted from [7]. *E2* estradiol, *FP* follicular phase, *LH* luteinizing hormone, *LP* luteal phase, *P4* progesterone

The menstrual cycle can be considered a vital sign [9–11] for female athletes of “reproductive age” who are not using hormonal contraceptives, pregnant, or nursing. The menstrual cycle is characterized by a series of regularly fluctuating concentrations of endogenous hormones, including E2, P4, LH, and FSH [7, 12]. The menstrual cycle consists of two basic phases, including the *follicular phase*, characterized by lower concentrations of E2 and P4, where E2 increases steadily and peaks before the LH surge/ovulation, and the *luteal phase*, characterized by higher concentrations of both E2 and P4 that decrease prior to a new cycle. The follicular phase and luteal phase are often separated by a surge in LH, which may be indicative of ovulation. In the research settings, and for clarity, in cycles of 21–35 days, the term “eumenorrhea” can be used to indicate a “textbook” menstrual cycle with a “correct” hormone profile consisting of four distinct phases, while the term “natural menstrual cycle” can be used when specific hormonal criteria are not being measured but cycle characteristics indicate a “normal” cycle [7] (for more specific definitions, including phases, see Fig. 1). Identification and reporting of these four distinct phases may be relevant in research but may not be feasible in practice where identification of bleeding and the LH surge are the most accessible markers to characterize menstrual cycle length and ovulatory status, respectively. Importantly, there is considerable intra- and inter-individual variation observed in the hormone profiles of non-pathological (“normal” or “natural”) menstrual cycles [13], demonstrating that a strictly “eumenorrheic” cycle is not necessarily the only acceptable cycle for health or performance. That is, ovulatory disturbance (anovulation), inadequate luteal phase, and/or small changes in cycle length that are transient and not clinically problematic may sometimes be observed. These subclinical disturbances in menstrual function may be adaptive [14, 15] and are generally not cause for immediate alarm, although repeated observations or marked changes in bleeding volume/pattern warrant medical consultation. Figure 1 provides more specific definitions for language used in the present article and should not be interpreted as a clinical screening tool.

A natural or eumenorrheic cycle (Fig. 1) starts with uterine bleeding in the early follicular phase that typically lasts for approximately 5 days [7, 11]. Uterine bleeding is often, but not always, associated with symptoms such as pain and cramping. Transient symptoms during the menstrual cycle that do not cause marked impairment are indicative of normal physiological premenstrual symptoms [16]. Prior to uterine bleeding, in the late luteal phase, ~ 7–10 days before a new cycle, up to 80% of women report at least one physical or affective symptom related to their menstrual cycle; however, most do not report marked impairment in their daily life [17]. Both menstrual and premenstrual symptoms can vary in severity and frequency between individuals as well as between menstrual cycles; as such, it is important to recognize that symptoms range from normal and relatively minor physiological symptoms to more problematic symptoms associated with premenstrual syndrome (PMS) and premenstrual dysphoric disorder (PMDD), which may disrupt daily life and for which standard clinical diagnostic criteria exist [16, 18]. PMS symptoms may include bloating, constipation, diarrhea, changes in mood, fatigue, irritability, and sleeping problems as well as muscle/joint pain (e.g., lower back) and headaches, which negatively affect the daily life of some ~ 15–20% of females [19]. The prevalence of PMS appears to be relatively high in the athletic population, with prevalence ranging from 49 to 60% in studies that adopted the gold standard method (prospective charting of symptoms) to assess PMS [20]. For some ~ 3–8% females, severe psychological symptoms that disrupt daily life are experienced during the premenstrual phase, a condition known as PMDD [21]. The prevalence of PMDD in athlete populations ranges from ~ 1 to 13% [22]. Where unpleasant physical and psychological symptoms are most common at the beginning (early follicular phase) and the end (late luteal phase) of the menstrual cycle, it is important to recognize that not all female athletes experience marked symptoms [23–26].

### Menstrual Disturbance/Dysfunction

An estimated 20% of footballers will present with menstrual disturbance/dysfunction [27] that may be classified as ovulatory disorders [28] or abnormal bleeding [29, 30]. Ovulatory disorders and abnormal bleeding are often accompanied by subclinical or clinical deviations from “normal” hormone profiles that may be characterized by suppression of the HPO axis resulting in lower concentrations of E2 and/or P4 [15] and a blunted or non-existent LH surge indicating anovulation, i.e., lack of ovulation (Table 1). Prevalence of menstrual disturbance/dysfunction is difficult to assess because definitions for menstrual cycle-related problems and assessment methods vary in the literature [20]. Nevertheless, a recent systematic review indicated primary amenorrhea in 7% of athletes, secondary amenorrhea in 16% of athletes, and oligomenorrhea in ~ 24% of athletes, while the most prevalent form of menstrual dysfunction identified was dysmenorrhea (painful menstruation), in ~ 32% of athletes [20].

**Table 1:** Terminology used to characterize patterns of menstrual disturbance/dysfunction and the potential implications of these characteristics on athletes

Term	Definition	Implications for athletes
Menstrual disturbances/dysfunction	*Ovulatory disorders and abnormal uterine bleeding* Ovulatory disorders are often suggested by the presence of abnormal uterine bleeding, which ranges from complete absence (amenorrhea) to infrequent or irregular menstruation, which may include heavy menstrual bleeding [28–30, 42]	May affect subjective as well as objective measures of performance and well-being [43, 44] Associated with LEA [31] where risk increases linearly as EA decreases [45], but a relationship between EA and menstrual disturbances is not consistent [46–48]. Is also associated with REDs [32, 49–51], Triad [52–56], and overtraining [57]
Amenorrhea	Absence of menstruation, suppressed HPO-axis function, i.e., consistently low E2 and P4 *Primary amenorrhea:* Failure of onset of menstruation by 15 years of age *Secondary amenorrhea:* Absence of menstrual cycle for > 180 days after at least one spontaneous menses	Amenorrhea indicates a lack of proper HPO-axis function, which may or may not be accompanied by dysfunction in other hormonal axes [31, 49]. This hormonal dysfunction may have direct and indirect implications for athlete health, wellness, and performance [58]
Anovulation (without amenorrhea) *Abnormal uterine bleeding – ovulatory (AUB-O)*	Failure to ovulate/detect an LH surge using urinary tests May occur sporadically in up to 1/3 of otherwise healthy cycles [59]; may be accompanied by consistently low E2 and P4 or luteal phase deficiency. Anovulation can be characterized by production of ovarian E2 without adequate P4, resulting in endometrial thickening without shedding or by inadequate ovarian E2, resulting in a thin endometrium [60]	Repeated observations associated with amenorrhea [36]
Infrequent menstruation *Oligomenorrhea*	Prolonged cycles > 35 days, can be ovulatory or anovulatory	Repeated observations may indicate ↑ risk for amenorrhea [61]
Luteal phase deficiency */*Inadequate luteal phase *Abnormal uterine bleeding – endometrial (AUB-E)*	Concentrations of P4 < 16 nmol·L^−1^ or 12.5 nmol/L [41] during the luteal phase; may occur in an intermittent and inconsistent manner. Indicates poor endometrial maturation due to inadequate P4 and/or a short luteal phase < 10 days [41, 62]. There is no agreement on criteria for diagnosing luteal phase deficiency [63]	Repeated observations may indicate ↑ risk for amenorrhea [61]
Dysmenorrhea	Painful menstruating	May ↓ subjective feelings, disabling symptoms, and disrupted physical activity [64]
Heavy menstrual bleeding (HMB)/menorrhagia and/or prolonged menstruation	Excessive menstrual blood loss that interferes with physical, social, emotional, and/or material quality of life and/or a menstrual period (bleed) lasting > 8 days. Heavy menstrual bleeding is more common in women with irregular cycles [65]	↑ risk for iron deficiency and anemia [66–68] May negatively affect perceived performance, decrease quality of life, and increased perceived stress [69, 70]
Frequent menstruation *Polymenorrhea*	Menstrual cycle of < 21 days. Possible but statistically uncommon [71]	Inconvenient. Frequent bleeding may indicate ↑ risk for iron deficiency and anemia
Intermenstrual bleeding/spotting (non-hormonal contraceptive use)	Spontaneous uterine bleeding that occurs between regular menstruation that is either cyclical occurring just before ovulation, due to a decline in E2 [72] and/or P4 withdrawal, which may be associated with luteal phase deficiency [63] or random. Intermenstrual bleeding is more common in women with irregular cycles [65]	Inconvenient. May influence subjective feelings and may be associated with, e.g., luteal phase deficiency/subtle menstrual disturbances/dysfunction [63]
Unscheduled bleeding or spotting (hormonal contraceptive users)	Uterine bleeding associated with the use of exogenous sex steroids for contraception. Common when commencing hormonal contraceptive use [73], especially if hormonal contraceptive use is commenced mid-cycle [74]	May influence subjective feelings/cause worry and may be an indicator to discuss contraceptive choices with a physician [73, 75]

For more specific information about ovulatory disorders and abnormal uterine bleeding, see [28, 29]. For depictions of hormone profiles for amenorrhea, anovulation, infrequent menstruation (oligomenorrhea), and luteal phase deficiency (inadequate luteal phase) see [31], and for recently updated terminology to describe abnormal bleeding in female athletes, see [42]. Please note that indicators and diagnostic criteria may vary depending on the source and that the current knowledge base regarding the implications of menstrual disturbance/dysfunction for athletes is limited

*E2* estradiol, *EA* energy availability, *HPO* hypothalamic-pituitary-ovarian, *LEA* low energy availability, *P4* progesterone, *REDs* Relative Energy Deficiency in Sport, *Triad* Female Athlete Triad

As mentioned, menstrual disturbance/dysfunction, including ovulatory disorders and abnormal uterine bleeding [28–30], appear to lie on a continuum characterized by varying degrees of HPO-axis (and potentially other hormonal axes) suppression [31]. HPO-axis suppression ranges from luteal phase deficiency to amenorrhea [28], while abnormal uterine bleeding can range from spotting and unscheduled intermenstrual bleeding to heavy bleeding [30]. Importantly, menstrual disturbance/dysfunction may be an overall indicator of endocrine function/dysfunction as it appears to be accompanied by disturbances in the hypothalamic-pituitary-thyroid axis and the hypothalamic–pituitary–adrenal axis, as well as the insulin-like growth-factor and growth-hormone axis [31, 32]. While problematic LEA is recognized as a relatively common cause of HPO-axis suppression in athletic populations [32], other non-pharmacological reasons for menstrual suppression also exist, such as physiological and/or psychological stress [33–35], gynecological illnesses [36, 37], pregnancy [38], and breastfeeding [39]. Dichotomization or isolation of stressors or other causes of ovulatory disorders and/or abnormal uterine bleeding may not, however, be justified, as the above causes of menstrual disturbance/dysfunction can work individually or synergistically to up- or down-regulate hormonal function [40]. Importantly, only a physician can properly diagnose menstrual disturbance/dysfunction, as coaches and even skilled members of the MDT may be ill-equipped to adequately address these medical issues. As part of medical evaluation, physicians may initially seek information on the following: days since previous menstruation, average length of cycle/regularity of cycle, or acute deviation from average length of cycle/regularity of cycle. Additionally, information about uterine bleeding patterns and duration, pain/dysmenorrhea, other symptoms, and their frequency or severity may be considered [30]. If available, information about possible anovulatory cycles (repeated lack of adequate LH surge) and/or inadequate luteal phase [negative urinary pregnanediol-3-glucuronide (PdG) test or short luteal phase] [41] may also be helpful. Ultimately, tracking the menstrual cycle can help to facilitate early identification of menstrual disturbance/dysfunction, which may be associated with additional symptoms that affect an athlete's health and well-being as well as performance.

### Hormonal Contraceptives

Approximately half of all elite female athletes use hormonal contraceptives [76], with combined oral contraceptives generally being the most common (up to 70% of hormonal contraceptive users). Recently, the use of hormonal (progestin-only) intrauterine devices (IUDs) including levonorgestrel as well as non-hormonal (copper) IUDs has increased [25]. The prevalence of hormonal contraceptive use across sports and countries varies [77, 78]. In the English Women’s Super League, 28% of players reported current hormonal contraceptive use, while 43% reported previous use. Combined hormonal contraceptives tend to be most popular (62% of total usage), followed by progestin-only hormonal contraceptives (38% of total usage) [77]. In Australian football codes (rugby league, rugby union/sevens, Australian football), a third of athletes reported current hormonal contraceptive use, of which combined hormonal contraceptives were most common [79]. Given the prevalence and potential for use of hormonal contraceptives in elite football, it is important to understand the mechanisms by which hormonal (and non-hormonal) contraceptives work before planning and implementing menstrual health monitoring.

**Fig. 2:**
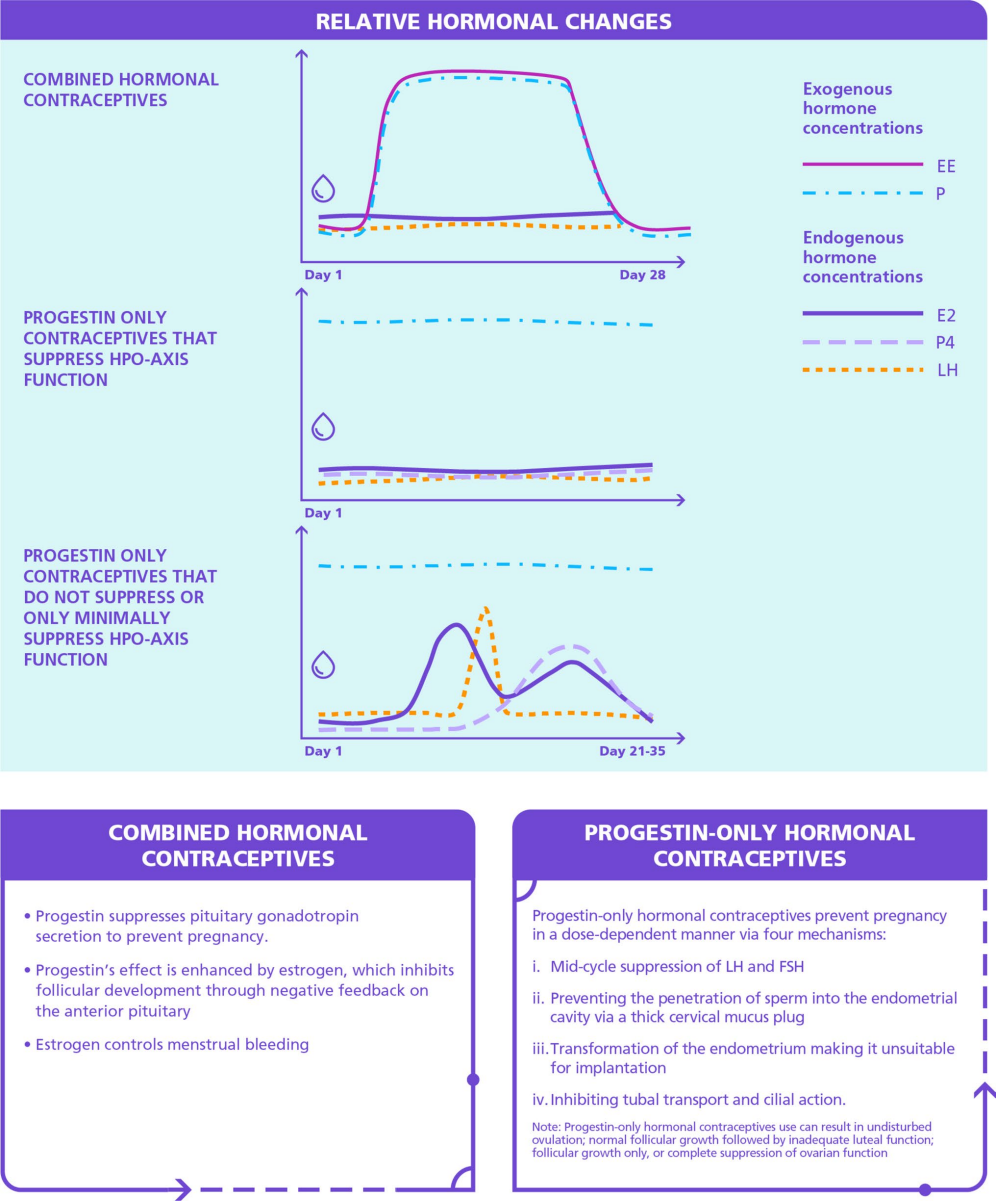
Hormonal contraceptive cycles. Endogenous hormones: estradiol (E2), progesterone (P4), and luteinizing hormone (LH); exogenous hormones: ethinyl estradiol (EE) and progestin (P). Informed by [94–96]. *FSH* follicle-stimulating hormone

Hormonal contraceptives utilize either a combination of estrogen (ethinyl estradiol, natural estradiol, or a derivative) and progestin (combined hormonal contraceptives), or progestin only. The purpose of these exogenous hormones, to a lesser or greater extent, is to suppress HPO-axis function (Fig. 2). Dose and delivery methods of hormonal contraceptives vary, but combined hormonal contraceptives generally include a 21- to 24-day “active” phase followed by 7–4 days of an “inactive” hormone-free or placebo phase (this applies to both oral contraceptives and the vaginal ring), while progestin-only hormonal contraceptives are continuously consumed or placed internally. The uterine bleeding that occurs in combined hormonal contraceptive users during the inactive, placebo, pill- or ring-free days is not considered true “menstrual bleeding,” although this so-called “withdrawal bleeding” is caused by a drop in exogenous hormone levels that causes the endometrium to shed, comparable to a “natural” menstrual cycle. Combined hormonal contraceptives and higher dose progestin-only contraceptives might be used for more extended periods of time to prolong HPO-axis suppression and prevent uterine bleeding and menstrual symptoms. Notably, when using low-dose progestin-only hormonal contraceptives such as hormonal IUDs and “mini-pills,” HPO-axis function can be maintained, or partly maintained, even in the absence of uterine bleeding [80, 81]. That is, in progestin-only hormonal contraceptive users, such as hormonal IUD and progestin-only pill users, the LH surge associated with ovulation may occur [82], whereas injectable formulas may fully suppress HPO-axis function [83]. Bleeding patterns in progestin-only users may require up to 3 months to become established and range from no bleeding to normal monthly bleeding [84]. Unpredictable or unscheduled uterine bleeding (including spotting) may occur with use of any hormonal contraceptive, although the mechanisms behind the bleeding are not fully understood [84, 85].

Hormonal contraceptives are used for several reasons beyond family planning in athletes, including mitigating symptoms of uterine bleeding and pain associated with menstruation as well as PMS/ PMDD, which are associated with ovulatory cycles [86]. Hormonal contraceptives can be used to control, plan, or suppress both “normal” uterine bleeding as well as heavy or abnormal (e.g., intermenstrual) uterine bleeding and are an excellent tool for preventing iron deficiency [87]. Importantly, current evidence does not indicate that hormonal contraceptives are performance enhancing or that they should be used for injury prevention.

Hormonal contraceptive use may be accompanied by adverse or unpleasant side effects. Most side effects are associated with the commencement of hormonal contraceptive use and diminish with continued use of the same formula and delivery method, typically within 3–5 months [88]. Side effects of hormonal contraceptives may include tender breasts, decreased libido, depressive symptoms, and mood swings [89] as well as unscheduled bleeding or spotting [80, 81, 84, 90]. Where hormonal contraceptives may be used to mitigate symptoms related to the menstrual cycle, hormonal contraceptive use does not necessarily eliminate all symptoms. That is, hormonal contraceptive users may experience adverse symptoms comparable to those associated with a natural menstrual cycle before, during, and after planned breaks from pill taking (or placebo) [23, 91]. While some studies have called attention to changes in subjective perceptions of performance (e.g., mental performance and force production) over the course of their hormonal contraceptive cycle [23], others have described unpleasant physical and psychological symptoms both at the beginning and the end of the hormonal contraceptive cycle (beginning of active phase and transition from active phase to inactive/placebo phase) [23, 24]. Importantly, comparable to the menstrual cycle, these studies indicate that not all females experience, or report, marked unpleasant physical and psychological symptoms over their hormonal contraceptive cycle. This highlights the importance of tracking symptoms and subjective feelings also in hormonal contraceptive users. Ultimately, hormonal contraceptives can significantly decrease physical and psychological symptoms as well as bleeding, which may result in a more positive overall situation that can facilitate quality training days. Furthermore, the plethora of formulations and developments in dosage and administration methods over the past decades has made it possible for more individualized approaches to prescription, which likely has increased compliance [86] as well as quality of life and satisfaction for users. Athletes and coaches should be aware that hormonal contraceptives are not the only way to treat or manage adverse symptoms related to the menstrual cycle. Athletes with concerns related to hormonal contraceptive use should seek advice from a physician/gynecologist. Players must be informed of indications, contraindications, options, and alternatives for hormonal contraceptive use and should be informed that the decision to use, or not use, hormonal contraceptives is theirs to make in consultation with their physician.

Briefly, non-hormonal IUDs are also available in the form of copper IUDs. Copper IUDs slowly release copper into the uterus thus rendering it hostile to sperm thereby preventing pregnancy. Copper IUDs do not influence HPO-axis function, although slight changes in bleeding patterns (generally increased bleeding) and changes to cycle length have been reported [92, 93]. Importantly, copper IUD users can experience observable menstrual disturbance/dysfunction. As such, tracking for copper IUD users can mimic that of naturally menstruating/eumenorrheic females or those with menstrual disturbance/dysfunction.

### Observing and Interpreting Changes

In individuals with natural cycles or established eumenorrheic cycles, changes in cycle characteristics such as a shortening (< 21 days) or lengthening of the cycle (> 35 days) for consecutive cycles; a repeated lack of LH surge [97] [and a lack of increase in estrone conjugate (E1C)]; repeated observations of low P4 (or PdG); repeated observations of short luteal phase; and cessation of bleeding should be addressed with a physician. For individuals with natural cycles, established eumenorrheic cycles, or hormonal contraceptive use, changes in uterine bleeding (changes in bleeding pattern and volume and/or spotting between cycles or during hormonal contraceptive use), and/or an increasing severity of symptoms or side effects should be addressed with a physician. It is, however, important to note that temporary and non-pathological changes in cycle characteristics can occur in naturally menstruating/eumenorrheic individuals as indicated in Sect. 2.1. For example, anovulation (indicated, e.g., by a lack of LH surge) can occur sporadically in healthy premenopausal women, as population-based research indicates that approximately a third of “normal” menstrual cycles may be anovulatory/present with ovulatory disturbances [59]. This underscores why it is important to monitor trends in menstrual health as well as overall health and performance rather than making hasty decisions (or diagnoses) based on a single measurement/observation. That is, in natural or established eumenorrheic cycles, a single observation of, e.g., anovulation (lack of LH surge) or inadequate luteal phase (short luteal phase or low P4/PdG) without any indicators of decreased performance or inadequate recovery does not warrant immediate cessation or modification of training. In contrast, an increase in severity of symptoms/side effects or uterine bleeding may warrant immediate medical attention. Proactive identification and treatment of subclinical menstrual disturbance/dysfunction is, however, desired because repeated observations may be a precursor to clinical menstrual disturbance/dysfunction that might be associated with decrements in health and performance [31, 98]. Furthermore, subclinical menstrual disturbance/dysfunction may already indicate that a player is not tolerating load caused by multiple stressors and is at a health and/or performance risk. In individuals with menstrual disturbance/dysfunction, tracking can help to identify further increases or decreases in the severity of adverse symptoms that can be addressed with the MDT or a physician. Likewise, tracking can help identify the return of “natural/eumenorrheic” cycle characteristics or confirm no change or worsening of identified problems. Athletes should be assured that singular measurements/observations may not be indicative of overall health/performance and should consult a physician with any concerns. When repeated observations of subclinical menstrual disturbance/dysfunction are made, a physician should be consulted to determine if there is a differential diagnosis (including, but not limited to, gynecological illnesses) and treat/advise the athlete accordingly [99].

The effects of LEA and physical and psychological load (multiple stressors) have been described in papers regarding the Female Athlete Triad [52, 53, 100], Relative Energy Deficiency in Sport (REDs) [32, 50, 51], or the Overtraining Syndrome [101, 102], all of which include mention of menstrual disturbances/dysfunction in individuals not using hormonal contraceptives. Unfortunately, the effects of LEA and physical and psychological load (multiple stressors) on combined or progestin-only hormonal contraceptive users have only briefly been addressed in the literature. Nevertheless, it is reasonable to suggest that hormonal contraceptive users are not immune to the effects of LEA and physical or psychological load. Current research indicates that hormonal contraceptives may maintain some level of already suppressed HPO-axis function during LEA and stress, but disturbances comparable to those observed in naturally menstruating females might occur in other hormonal axes ([103] and [104]). Importantly, while hormonal contraceptive users may encounter challenges related to under-fueling, overtraining, and psychological stress, their “hormonal cycle”/uterine bleeding pattern is unlikely to indicate problems in the way that menstrual cycle characteristics in individuals not using hormonal contraceptives can. Additionally, it is important to understand that athletes experiencing menstrual disturbance/dysfunction might be prescribed hormonal contraceptives to help mitigate or “manage” symptoms, but in some situations, the underlying issue, possibly LEA or psychological stress, may not have been recognized and addressed, thus the underlying issues may remain and might even become more problematic. As such, it is essential for athletes, coaches, and MDTs to understand how hormonal contraceptive use can mask menstrual disturbance/dysfunction, and it may be necessary for coaches and MDTs to assist athletes in developing specific strategies to ensure energy availability, adequate recovery, and stress management.

**Fig. 3:**
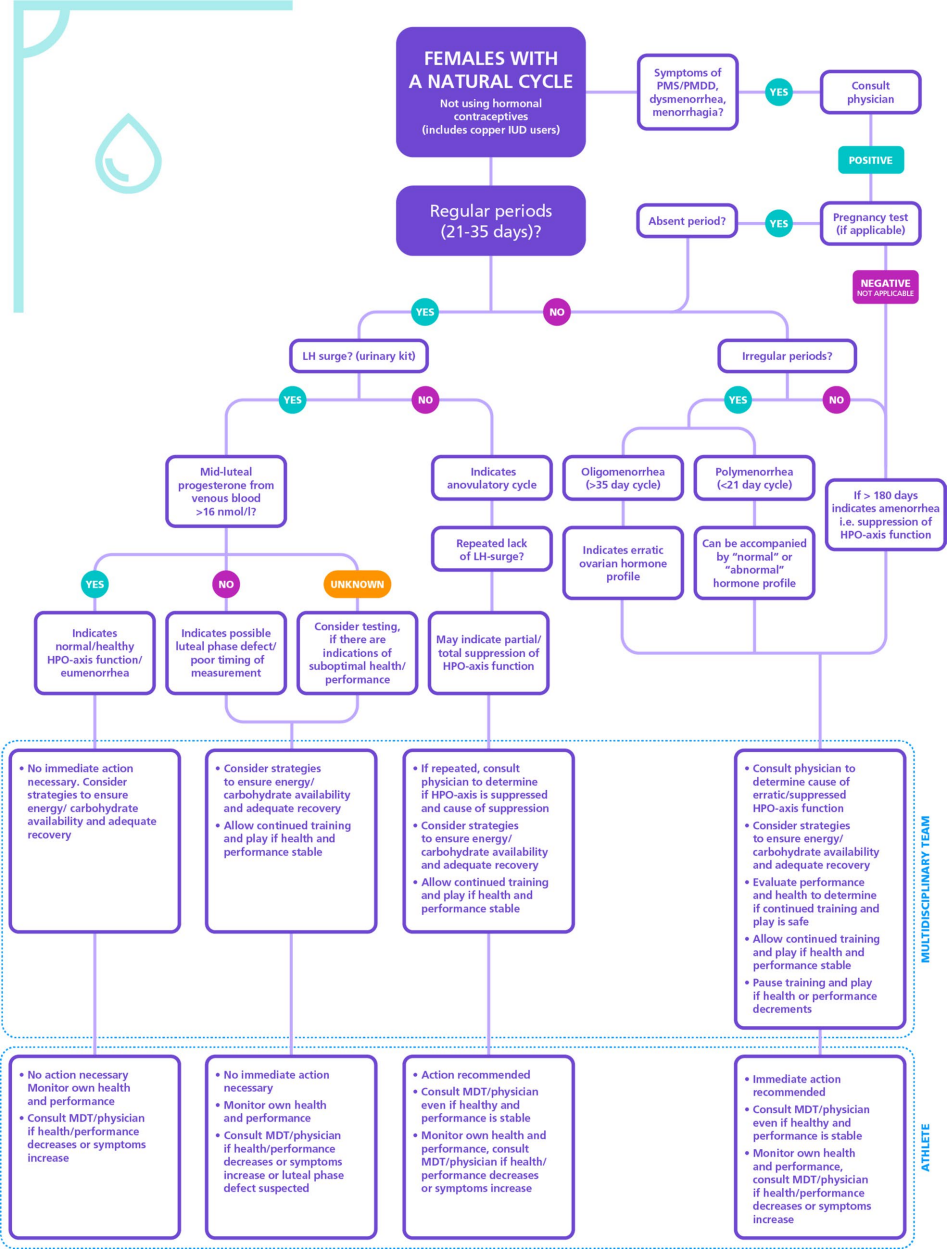
Decision tree to guide athletes and MDTs regarding HPO-axis function in females with natural cycles (i.e., not using hormonal contraceptives). This decision tree is for educational purposes and should not be used for clinical purposes or in place of medical advice from a physician. If the athlete is only able to track bleeding, cycles of normal length require no immediate action, and training can continue if health and performance are stable. As with all athletes, strategies to ensure energy/carbohydrate availability and adequate recovery should be considered and a physician should be consulted if health/performance decreases or symptoms increase. *HPO* hypothalamic-pituitary-ovarian, *IUD* intrauterine device, *LH* luteinizing hormone, *MDT* multidisciplinary healthcare team, *PMDD* premenstrual dysphoric disorder, *PMS* premenstrual syndrome

**Fig. 4:**
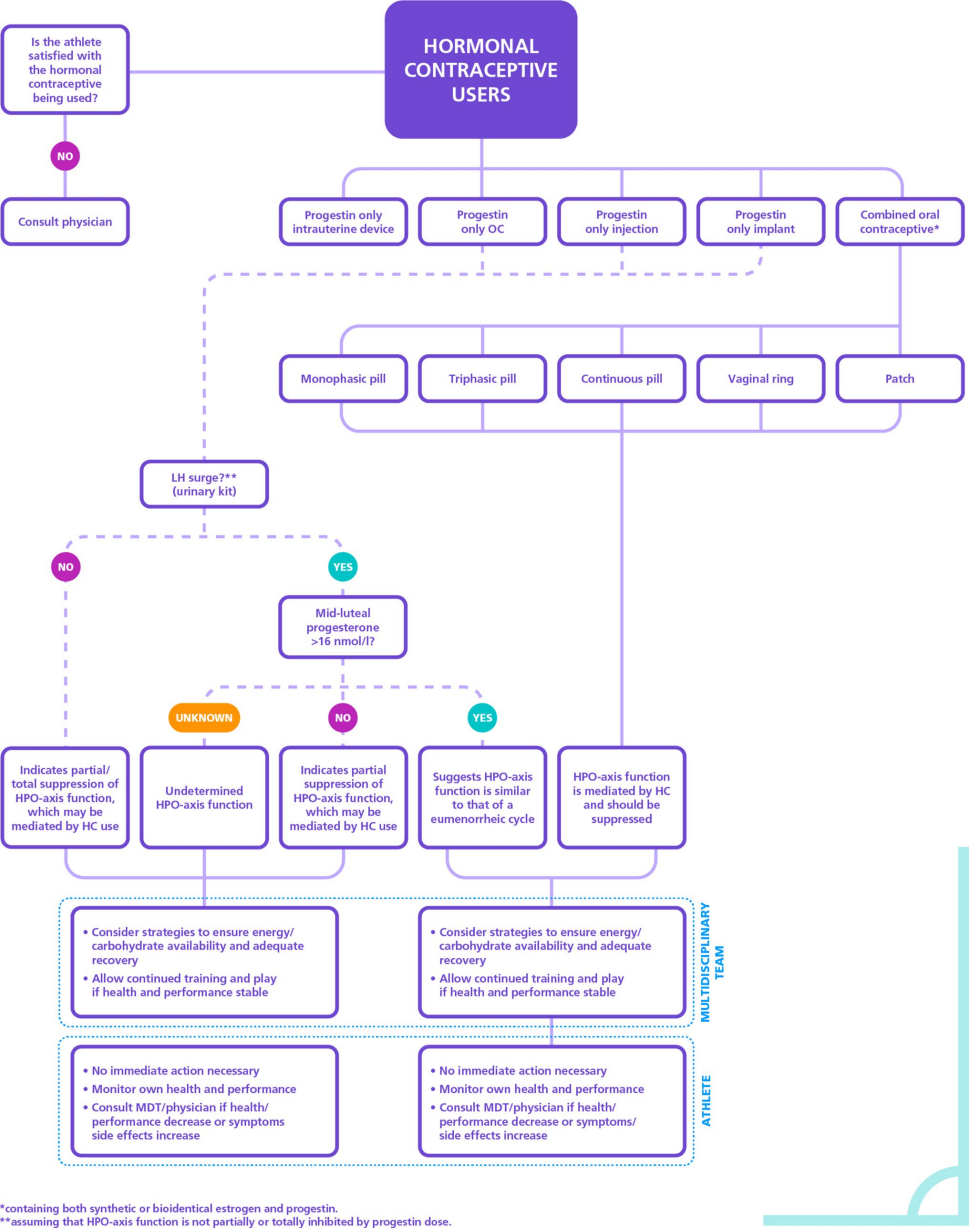
Decision tree to guide athletes and MDTs regarding HPO-axis function in females using HCs. *Dashed lines* indicate a proposed tracking method for athletes using progestin-only methods that do not fully suppress HPO-axis function. Notably, the current knowledge base regarding tracking the LH surge or progesterone levels in HC users is limited. This decision tree is for educational purposes and should not be used for clinical purposes or in place of medical advice from a physician. *HC* hormonal contraceptive, *HPO* hypothalamic-pituitary-ovarian, *LH* luteinizing hormone, *MDT* multidisciplinary healthcare team, *OC* oral contraceptive

In Figs. 3 and 4, we provide decision trees that can be used as tools for athletes and MDTs to interpret HPO-axis function, including a brief interpretation of results for MDTs and actions for athletes. These decision trees are educational and should not replace diagnosis/treatment by a physician with knowledge of sports and menstrual health, while training, recovery, nutrition, and general well-being should always be considered while assessing player health. Other tools for evaluating menstrual health have been published elsewhere [105, 106]. While tracking urinary, salivary, or serum hormones is not a well-established method in progestin-only hormonal contraceptive users, Fig. 4 suggests that this might be possible. As previously mentioned, HPO-axis function can be maintained in low-dose progestin-only hormonal IUD and pill users [82] even in the absence of uterine bleeding [80, 81], although HPO-axis function is likely to be suppressed in those using injections [83]. Unfortunately, in progestin-only users, it is difficult to differentiate whether HPO-axis suppression is attributed to exogenous progestin, LEA/multi-stress, or some other factor. At present, the benefits of tracking urine-based hormonal variables in this population are unclear.

## Monitoring Menstrual Health in Football

**Fig. 5:**
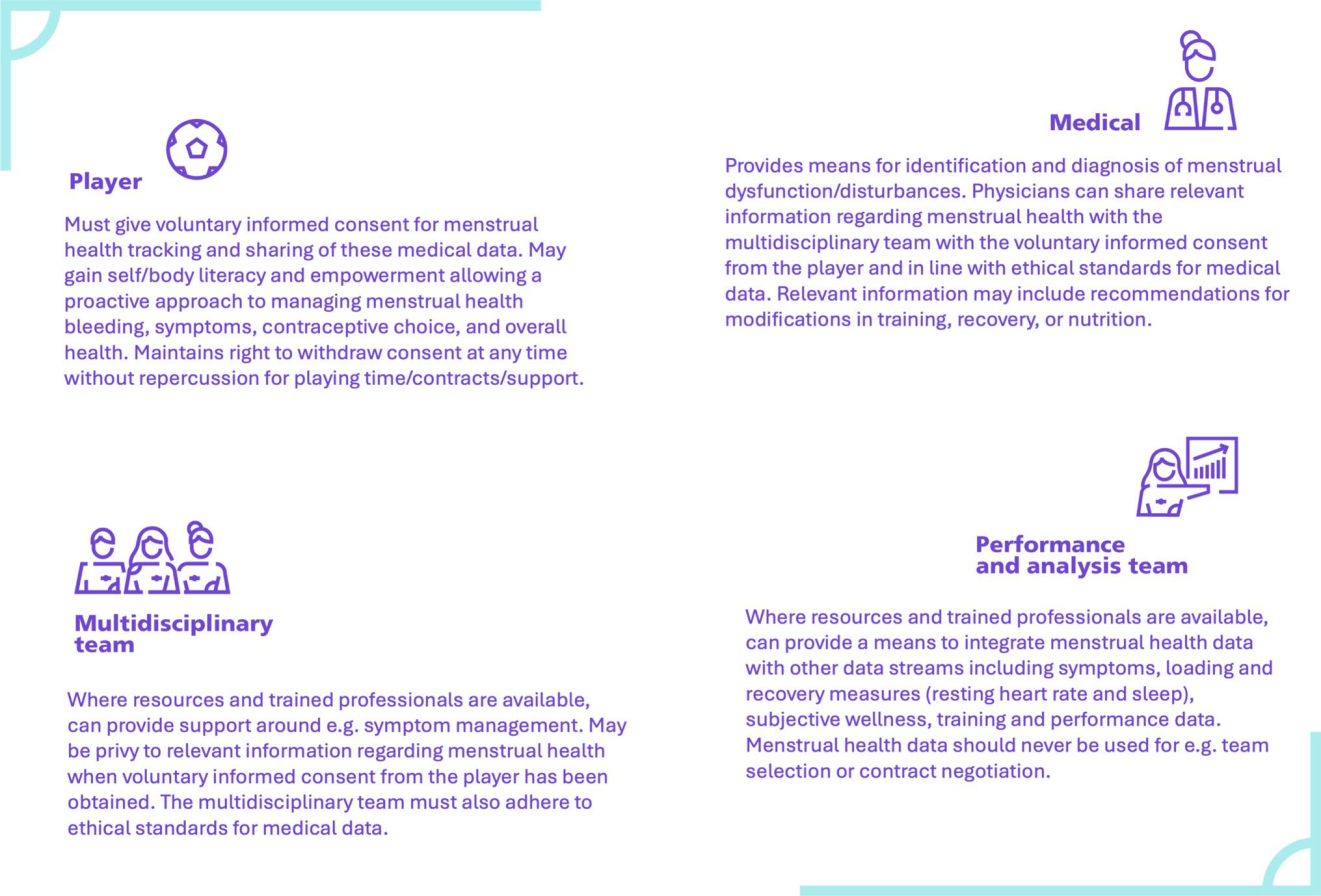
Roles of players, the multidisciplinary, medical, and performance analysis teams in menstrual health tracking and monitoring

Monitoring overall fitness/performance and health is already part of many routines in established teams that can be even more impactful when integrated with menstrual health (Fig. 5). Tracking the menstrual cycle of female athletes has become an aspect of health management and even performance optimization [107, 108], while tracking menstrual disturbance/dysfunction (ovulatory disorders and abnormal uterine bleeding) and hormonal contraceptive cycles has received less attention. It has been established that both E2 and P4 have functions beyond reproduction within the body, influencing muscle, nervous, epithelial, and connective tissues while playing a role in several physiological processes, including metabolism, respiration, cardiovascular function, immunity, gastrointestinal, and genitourinary function as well as neural functions, including the autonomic nervous system and cognition (see, for example, [109, 110]). As such, it is reasonable to infer that HPO-axis function and dysfunction might influence female athlete health, well-being, and performance. At the same time, it is important to recognize that a single hormone or hormonal measure does not confirm or dispute the absence or presence of menstrual or overall health. Thus, players should be referred to their MDT and/or a physician outside of their organization to address health concerns.

No one-size-fits-all approach for monitoring menstrual health or tracking hormonal cycles exists. Where capturing a “eumenorrheic” hormone profile may be important for understanding the possible influence of E2 and P4 concentrations, and their ratios, on performance [7, 111], particularly in research settings, using rigorous testing protocols to regularly confirm “eumenorrhea” in a field setting may not be practical or desired. As such, tracking basic menstrual or hormonal contraceptive cycle characteristics (cycle length, uterine bleeding patterns) and symptoms/side effects, as well as subjective feelings regarding readiness and performance can be the initial proactive step towards managing overall health and well-being while supporting female athlete development. Additional tools/methods, such as hormone analysis from, e.g., blood, urine, or saliva can be implemented when warranted and when resources and qualified personnel are available. Data from tracking cycles, regardless of menstrual status or hormonal contraceptive use, can help individual athletes, coaching staff, and MDTs understand the changing rhythms of each athlete’s body, improving body literacy and thus facilitating the identification of situations where communication and follow-up may be necessary [112]. It is worth reiterating that tracking cycle characteristics alone can provide valuable information that can be used to identify menstrual disturbance/dysfunction while helping to inform athlete–physician decisions about the possible use of contraceptives, medications, and even dietary strategies/supplements. Tracking also allows athletes, coaching staff, and MDTs to be proactive and prepared with, e.g., appropriate methods for managing/mitigating/treating pain, bleeding, and symptoms/subjective feelings [113]; providing sanitary products; and preparing plans for referring athletes to the appropriate healthcare providers (in cases where an organization’s MDT is not able to address concerns adequately).

While there is currently only limited scientific evidence linking menstrual disturbance/dysfunction to decreased performance or blunted training adaptations, it is reasonable to suggest that menstrual disturbance/dysfunction eventually negatively impacts these parameters [31, 98, 114, 115], although effects may be indirect (see Table 1 “Implications for athletes”). At a minimum, the identification of amenorrhea/oligomenorrhea and abnormal uterine bleeding may be relevant for individual athletes and their physicians [60, 116] and, by extension, MDTs and coaching staff, as they may indicate a need for medical follow-up; adjusted training; prioritization of rest, recovery, or nutrition; consideration of mental health; and/or return-to-play planning. During possible treatment of menstrual disturbance/dysfunction by a physician, and in conjunction with addressing possible under-fueling, overtraining, or psychological stress within an MDT, tracking cycle characteristics and symptoms can be used to monitor progress related to both menstrual and general health.

As previously stated, monitoring menstrual health should not be exclusive to athletes with natural or eumenorrheic cycles or those with menstrual disturbance/dysfunction. Tracking cycle characteristics in hormonal contraceptive users does, however, require some knowledge of the different types (type, dose, delivery method) of hormonal contraceptives available, as described above in Sect. 2.3. Tracking in combined hormonal contraceptive users can be planned in accordance with active and inactive pill-taking or breaks from vaginal ring use, while establishing a cycle in progestin-only users may be more difficult if regular bleeding is absent. Progestin-only IUD and low-dose pill users might be able to identify the LH surge and/or and mid-luteal P4, but this is not a well-established approach and requires further research. Nevertheless, comparable monitoring of menstrual health by tracking hormonal contraceptive cycle characteristics and related symptoms can be implemented in athletes using hormonal contraceptives.

In order to generate meaningful data and facilitate timely referrals to physicians or MDTs, regular tracking of menstrual and hormonal contraceptive cycles for a minimum of 3 months (or three cycles) is needed to establish a baseline [7], although additional time/cycles may be needed in cases of menstrual disturbance/dysfunction and commencing, discontinuing, or changing hormonal contraceptives [84]. Characteristics of menstrual function (overall cycle length, length of the luteal phase, estimated day of ovulation, duration of menstrual flow, menstrual intensity, and cervical mucus) may be altered for at least 2 cycles after cessation of hormonal contraceptive use [117] or even longer after injectable progestin-only use [83]. Establishing and understanding “normal” patterns in an athlete should evolve over time; thus, it is worth considering longer-term tracking of cycle characteristics, especially if comparing pre- to in-season patterns is desired.

Tracking can be commenced at any time, for example, when the athlete is well-rested and healthy, or even when disturbance/dysfunction is suspected. After establishing a baseline, tracking can be continuous or periodic. Periodic tracing can be commenced after marked shifts in training volume or intensity or modifications in nutrition and recovery strategies. Once again, even basic cycle characteristics, symptoms and side effects, and subjective feelings can provide valuable information to coaching staff and MDTs in the absence of more sophisticated measures, thus providing an opportunity to be proactive regarding menstrual health. Importantly, isolated, infrequent, or sporadic testing of menstrual cycle characteristics or hormone concentrations provides only limited information to athletes, coaches, and MDTs, suggesting that basic characteristic tracking should be consistent, while more sophisticated methods can be used less frequently and/or with guidance from a physician when menstrual disturbance/dysfunction is suspected, as described below.

Tracking cycles as part of health and performance monitoring can be started at any time during an athlete’s career including during/after puberty in junior athletes but should always commence with guidance and education about menstrual health (for athletes and parents) to promote voluntary and informed buy-in and open dialog [108]. For example, younger athletes, parents, coaching staff, and MDTs need to understand the physiology of puberty and the expected time course of “stabilization” of the menstrual cycle after menarche, as menstrual cycles may be irregular through adolescence [118]. Likewise, in more mature athletes, the “destabilization” that occurs in the menstrual cycle through pre- and perimenopause [65] is relevant. These transitions, those occurring when starting/stopping hormonal contraceptive use and those occuring with pregnancy, miscarriage, the postpartum (lactating) condition, menopause, and various illnesses are also important considerations, as these may impact tracking outcomes. Both younger and more mature athletes should be educated regarding menstrual disturbance/dysfunction and hormonal contraceptive use. Tracking may be continued over career and lifespan, but athletes, coaching staff, and MDTs need to consider what methods are practical, feasible, and purposeful. It is also worth considering what degree of tracking is appropriate without medical advice in younger/junior athletes for whom consent may need to be obtained from parents. Finally, teams/organizations implementing menstrual health tracking methods must be mindful of the possible added stress and burden for athletes of all ages in terms of collecting data and interpreting results.

### Tools for Tracking Hormonal Cycles

Several tools and methods have been developed or adopted to support athletes, coaches, and MDTs in their endeavor to track components of menstrual health. Naturally, each tool comes with its own benefits and challenges. From traditional paper and pencil recording to more sophisticated digital applications and biochemical measures, the range of tools available reflects the different needs and preferences of athletes as well as the resources available to implement these tools. The simplicity and accessibility of manual recording methods such as paper and pencil contrast with the detailed insights offered by technological solutions that can monitor and integrate additional physiological indicators such as basal body temperature, cervical mucus consistency, and hormone levels measured from blood, urine, or saliva. The choice of tracking method is often a trade-off between sensitivity and feasibility, which should specifically consider the athlete’s lifestyle, training schedule, and personal comfort level.

As research in this field continues to develop, so do the tools. Tools are becoming easier to seamlessly integrate into the daily routines of athletes and help provide a more comprehensive picture of training and recovery as well as overall health and performance. The following sections will describe tools for tracking menstrual health, including a brief description of their potential benefits and limitations. It should be noted that regular (i.e., year-round) tracking of serum hormone concentrations in a healthy athlete, and in the presence of consistently normal cycle characteristics, may not be warranted as the value of this additional information may be limited. If cycle characteristics indicate the presence of menstrual disturbance/dysfunction or there are other symptoms of underperforming or illness, the athlete should be referred to a physician for follow-up testing, which may include some of the methods/tools herein. Aside from collection of blood samples via venipuncture and assessment of follicles via transvaginal ultrasound, the latter of which requires medical justification, data collection can, and should, be completed by the player themself.

Table 2 describes what characteristics might be tracked in different hormone profiles, while Table 3 summarizes the general basic and digital tools available for tracking menstrual and hormonal contraceptive cycles, including methods for monitoring sex steroids (E2 and P4 or their derivatives) from blood, urine, or saliva. These methods can be applied when tracking natural/eumenorrheic menstrual cycles as well as when tracking menstrual disturbance/dysfunction or hormonal contraceptive cycles; however, interpretation of these data requires an understanding of the cycle being tracked as well as knowledge of how to interpret and utilize results for the benefit of the athlete. Tracking should be done with clear objectives regarding athlete health and performance and not just for the sake of tracking. Ultimately, these data can be used to contextualize other health and performance data while providing valuable information about an athlete’s health to MDTs. In the absence of access to tools for analyzing salivary, urine, or blood samples, tracking uterine bleeding (for cycle length) and adverse symptoms, while avoiding assumptions related to menstrual cycle phase, can still be considered.

**Table 2:** Tracking in athletes with different hormone profiles

Eumenorrheic/naturally menstruating (and copper IUD)	Menstrual disturbance/dysfunction	Combined hormonal contraceptives			Progestin-only hormonal contraceptives
Cycle can be established by monitoring basic/ “essential” menstrual cycle characteristics as well as urine/salivary/blood-based measurements	May be difficult to establish a cycle without uterine bleeding Working to identify an LH surge (E1C or PdG) is secondary to addressing root cause(s) of the dysfunction with a physician	Cycle is established by start of active phase (pill taking or insertion of vaginal ring) vs. placebo/breaks Not possible to identify menstrual disturbance/dysfunction			May be difficult to establish cycle in females without uterine bleeding but possible to try Difficult to identify “menstrual” disturbance/dysfunction unless a baseline has been established while using progestin-only hormonal contraceptives
*Essentials for tracking* Relatively simple tools that require only basic education for interpretation of results Can be used/assessed regularly					
Age of menarche					
Date of last menstrual period		Date of starting active phase and duration of use (21–24 “active” days vs. prolonged use)			Date of starting active pills, IUD, injection, or patch use and duration of use
Menstrual cycle length/regularity of bleeding					
Days of bleeding/presence of abnormal bleeding		Days of bleeding/presence of abnormal bleeding			
Use of menstrual hygiene products and description of flow (e.g., light, moderate, heavy) to determine volume of blood loss		Use of menstrual hygiene products and description of flow (e.g., light, moderate, heavy) to determine volume of blood loss, although flow should be mitigated with hormonal contraceptive use			
Pain associated with menses, including associated symptoms (nausea, diarrhea, fatigue, etc.) and impact on daily life (school, work, activities, performance)		Pain associated with bleeding, including associated symptoms (nausea, diarrhea, fatigue, etc.) and impact on daily life (school, work, activities, performance), although these should be mitigated with hormonal contraceptive use			
Symptoms associated with menstrual cycle in general and impact on daily life (school, work, activities, performance)	Symptoms associated with cycle in general and impact on daily life (school, work, activities, performance)	Symptoms and side effects associated with hormonal contraceptive use and impact on daily life (school, work, activities, performance)			
*Extras for tracking* May provide valuable information, but requires advanced education and resources Samples should be collected by the player themself (urine, saliva) or by a qualified professional (blood samples) Should be used with discretion					
LH surge (with or without E1C)		-			LH surge (with or without E1C)^a^
Luteal phase P4 or PdG		-			Luteal phase P4 or PdG^a^
(Phase-based) sex hormone measurements and/or other biomarkers					
*Extras for research or if medically necessary*					
May provide clinically valuable information, but must be collected by qualified medical personnel Requires advanced education and resources Should be used with medical discretion					
Transvaginal ultrasound to determine ovulation		-			Transvaginal ultrasound to determine ovulation^b^

Note: “Essentials” can be used daily, while “extras” should only be used with clear goals. Use of “extras” should, ideally, be preceded by adequate education of the MDT, physician, and athlete and should only be used when voluntary informed consent has been received from the athlete

*E1C* estrone conjugate, *HPO* hypothalamic-pituitary-ovarian, *IUD* intrauterine device, *LH* luteinizing hormone, *MDT* multidisciplinary healthcare team, *P4* progesterone, *PdG* pregnanediol-3-glucuronide

^a^Urine (saliva and blood)-based tracking is not an established method in progestin-only hormonal contraceptive users, although these methods could, in theory, be used in individuals who have maintained HPO-axis function during progestin-only hormonal contraceptive use

^b^In individuals who have maintained HPO-axis function during progestin-only hormonal contraceptive use, transvaginal ultrasound may be appropriate for assessment of ovulation

**Table 3:** Tools for tracking menstrual and/or hormonal contraceptive cycles

Tracking method	Benefits	Challenges	Sensitivity/reliability	Feasibility
*Basic methods*				
Pencil and paper: Retrospective tracking	Non-invasive and can gather comprehensive data on menstrual and hormonal contraceptive history, symptoms, and side effects	Relies on the athlete’s recall and diligence in recording; may not capture all relevant physiological information. Self-reported data may be subject to bias and inaccuracies; not all available questionnaires are validated in athletes	Low reliability, as it relies on subjective reporting Reasonable reliability if teams are able to effectively use validated questionnaires Relatively low sensitivity unless athlete recall and body literacy is high	Highly feasible, as it can be implemented easily with minimal cost, but requires careful design to ensure data quality Need for validated questionnaires in different languages
Pencil and paper: Prospective tracking	Non-invasive, simple method to gather comprehensive data on menstrual and hormonal contraceptive characteristics, symptoms, and side effects	Relies on the athlete’s diligence in recording; may not capture all relevant physiological changes. It requires the athlete to regularly transfer the data to an expert for “analysis”	Moderate reliability, as cycle characteristics, symptoms/side effects can provide valuable information regarding cycle characteristics Moderate sensitivity if tracking is consistent and body literacy is high	Highly feasible (easy to implement) and cost-effective Requires that athletes are committed to regular tracking and that athletes/teams are able to interpret the results
Basal body temperature (BBT)	*Manual:* Non-invasive; could indicate ovulation when a consistent increase BBT is observed, possibly allowing for identification of the luteal phase *Wearable:* Non-invasive; could indicate ovulation when a consistent increase in BBT is observed, possibly allowing for identification of the luteal phase	*Manual:* Requires daily measurements, typically taken at the same time each morning before any activity, which can be logistically challenging. Requires several cycles of tracking to establish a baseline *Wearable:* Requires consistent use, access to electricity for charging device; logistically easier than manual measurements. Requires several cycles of tracking to establish a baseline	Low reliability and sensitivity. Assuming a “eumenorrheic” cycle BBT can be used for tracking, but this method is *not effective for preventing pregnancy* as the increase in BBT happens after ovulation when concentrations of P4 increase. This method is *not reliable* for individuals with menstrual dysfunction or those using combined hormonal contraceptives but could be applied in progestin-only hormonal contraceptive users with maintained HPO-axis function	Feasible with proper education and adherence to consistent measurement practices but may be affected by factors like illness or disrupted sleep
Cervical mucus	Non-invasive; could indicate ovulation	Requires education to accurately interpret changes and may be influenced by factors like infections or sexual activity. This method is not effective in hormonal contraceptive users	With proper education, can be a reliable method to track fertility/ovulation [119, 120]. Cervical mucus reflects changes in E2 and pP4 levels; however, the interpretation of the changes is subjective	Feasible with proper education and adherence to consistent measurement practices but not recommended due to very low reliability and sensitivity
*Digital or “smart” tools*				
Online ovulation calculators	Non-invasive	Relies on the athlete’s diligence in recording; may not be designed to capture all relevant information. May be subject to bias and inaccuracies present within the online calculator No published methods for identifying appropriate and accurate calculators for athlete needs	Low reliability and sensitivity, as calculators rely on averages to predict the timing of ovulation and menstruation	Feasible for athletes/teams with access to the internet
Smart phone applications	Non-invasive	Relies on the athlete’s diligence in recording; may not be able to capture all relevant physiological changes. May be subject to bias and inaccuracies present within the application Data ownership is necessary to consider. Guidance included in applications may not be evidence-based or align with medical/research standards	Moderate reliability, as cycle characteristics and symptoms/side effects can provide valuable information regarding cycle characteristics. It should be noted that many applications will provide only a limited list of symptoms When combined with urinary hormone measures, algorithms based on the mid-cycle LH surge measured by over-the-counter urine tests appear to classify a higher proportion of anovulatory cycles when compared with algorithms that use serum P4 measurements from the luteal phase [121]	May be more accessible in better resourced countries Recommendations within the applications may not entirely be evidence-based [122]
Wearable devices (smart watches, rings)	Non-invasive Relies on indirect markers of menstrual cycle including heart rate variability and BBT [123, 124]	May not capture all relevant physiological changes; must be regularly used and are often connected to a specific smart phone application or applications Data ownership is necessary to consider. Guidance included in applications may not be evidence-based or align with medical/research standards	Variable reliability and sensitivity as interpretation of gathered data influenced by device-specific algorithms. Can identify fertile window, but research is limited, and errors in prediction have been reported [125]	Maybe more accessible in better resourced countries
*Hormonal measurements (blood, urine, saliva)*				
Ovulation kits (LH surge)	Can provide insights into the ovulatory phase via LH surge and help with understanding individual hormonal fluctuations	May require frequent testing of ~ 5–10 days per cycle (same time of day), which can be cumbersome and intrusive	Moderate reliability and sensitivity, although this may vary by kit used. Relies on the detection of LH surge, which may not perfectly correlate with actual ovulation and may be missed due to measurement timing	Feasible with the use of LH-surge kits, but cost and user compliance can be barriers
Ovulation kits (LH surge including E1C)	Can provide insights into the ovulatory phase via LH surge and estrogen concentrations and help with understanding individual hormonal fluctuations	May require daily testing of ~ 5–10 days per cycle (same time of day), which can be cumbersome and intrusive	Moderate reliability and sensitivity, although this may vary by kit used. Relies on the detection of increasing E1C and the LH surge, which may be missed due to measurement timing	Feasible with the use of LH and E1C combination tests, but cost and user compliance can be barriers
Serum blood, urine, or saliva samples for estrogen and P4	*Blood:* Invasive to obtain but has the potential to offer a more precise understanding of hormonal concentrations *Urine:* Minimally invasive but has the potential to offer a more precise understanding of hormonal concentrations *Saliva:* Minimally invasive but has the potential to offer a more precise understanding of hormonal concentrations	*Blood:* Requires (repeated) venipuncture and comes with a small risk for infection. Requires trained and qualified healthcare personnel for sample collection and analysis – may not be practical over a prolonged period of time. Samples require proper storage and transport before analysis *Urine:* Timing of measurements may be critical, and interpretation of results is specific to kits and reagents used. Interpretation requires education *Saliva:* Timing of measurements may be specific, and the interpretation of results is specific to kits and reagents used. Interpretation requires education	*Blood:* Relatively reliable/sensitive, but results must be contextualized, e.g., to menstrual cycle phase and consider possible hormonal contraceptives use. Testing is not necessarily warranted without clinical reasons *Urine:* Relatively reliable/sensitive, but results must be contextualized, e.g., to the menstrual cycle phase and consider possible hormonal contraceptives use. Thresholds for kits and their interpretation must be understood. Testing can commence without clinical reasons, but results should be interpreted with caution *Saliva:* Reliability and sensitivity affected by the rapid fluctuations in salivary concentrations of sex steroids [126] and collection method [127]	Less feasible for regular tracking due to the need for clinical intervention (blood), specific timing (blood, urine, saliva), and associated costs (blood, urine, saliva) Cost, user compliance, and time burden can be barriers (blood, urine, saliva)
Transvaginal ultrasound	Considered the gold standard for determining ovulation and can be used to assess endometrial thickness	Invasive [128]. May cause psychological distress in some populations and introduces potential for inadvertent boundary transgression [129]. Requires expensive equipment as well as trained and qualified healthcare personnel for data collection as well as a physician to interpret data and make diagnoses. Transvaginal ultrasound is not practical to use for “confirmation” of ovulations over a prolonged period of time. Necessitates appropriate cleaning and disinfection of the transducers between the procedures [130] and appropriate use of transducer cover materials [131]. Using ultrasound without medical indication can be considered ethically unjustifiable [132]	Considered the gold standard for determining ovulation and can be used to assess endometrial thickness. There are limitations in this gold standard related to the need for subjective interpretation of ovaries’ ultrasonic morphology [128]	Not feasible for regular tracking due to the need for clinical intervention, specific timing, and associated costs (equipment and medical expertise) Individuals undergoing transvaginal ultrasound may prefer a chaperone be present if the technician is male [133]

Please note that this table is a simplified summary and the actual methods may have more nuanced benefits and challenges. It is important to consider individual differences among athletes when choosing a tracking method. In this table, reliability reflects the degree of error in the method and sensitivity reflects the ability of the tool to identify small but possibly meaningful changes, as assessed by the authors of the paper who are experts in the field. Please note that tracking methods are intended to characterize menstrual and hormonal contraceptive cycles, and these methods are not being endorsed or suggested for preventing pregnancy. Some of these tools can be used for diagnostic purposes, but diagnoses should only be made by a physician

*E1C* estrone conjugate, *E2* estradiol, *HPO* hypothalamic-pituitary-ovarian, *LH* luteinizing hormone, *P4* progesterone

### Tracking Hormones “in Field Settings”

All athletes can potentially benefit from tracking subjective feelings, including those regarding mood and physical symptoms, alongside tracking of menstrual and hormonal contraceptive cycle characteristics. Tracking of basic symptoms and subjective feelings alongside overall health and fitness/performance can be the same for athletes with natural/eumenorrheic cycles, athletes with menstrual disturbance/dysfunction, and/or hormonal contraceptive users (Table 2). Where tracking basic cycle characteristics and subjective feelings can be done relatively quickly and with minimal effort or equipment, regular self-testing of urine or saliva and frequent blood draws can be time-consuming, expensive, and stressful. Moreover, methods for determining ovulation such as transvaginal ultrasound are invasive and should only be used when medically necessary [128, 132]. Importantly, signs of anovulation, a prolonged cycle, or signs of luteal phase defect (also known as inadequate luteal phase) from, e.g., urine tests may cause worry/stress even if they are not absolute indicators that something is “wrong.” This underscores the importance of tracking consecutive cycles, educating athletes about menstrual disturbances/dysfunction, and ensuring that referral processes are established if an athlete is concerned about their health.

In the field setting, pencil and paper, calendar, or application-based tracking can be combined with testing/screening hormonal concentrations using over-the-counter urinary hormone kits rather than gathering and analyzing blood samples. The combination of calendar-based counting and tracking of the urinary LH surge can help identify subclinical menstrual disturbances [134], although inclusion of a mid-luteal phase blood sample for the determination of P4 concentrations is more accurate [135]. Unfortunately, regularly collecting and analyzing blood samples for determination of hormone concentrations is not likely to be feasible or practical for most athletes/teams [136]. As such, athletes, coaches, and MDTs must determine if additional hormonal data (from urine/saliva) might be useful or meaningful if basic cycle characteristics fall within the normal range and athlete health/performance is at the expected level.

In a clinical setting, transvaginal ultrasound is the gold standard used to detect the growth and disappearance of a follicle to confirm ovulation and a eumenorrheic cycle, although it relies on the subjective interpretation of ovaries’ ultrasonic morphology [128]. Transvaginal ultrasound is invasive [128, 132] and by no means practical or necessary to implement in a sporting environment such as football. Measuring the LH surge (a surrogate measure for ovulation) from urine has gained popularity in recent years both in research and in practice and can be performed privately at home. While LH-surge kits, when used regularly by players, may be relatively reliable for predicting ovulation, indicative of normal menstrual function, athletes and MDTs need to understand the nuances and potential limitations of these tests. First, there is marked variability in LH-surge duration, configuration, and amplitude [137, 138], as eloquently illustrated in Colenso-Semple et al. [139] using data from Dam et al. [140]. Indeed, studies suggest that up to 18% of females experience an LH surge in the absence of ovulation, whereas premature LH surges, i.e., an LH surge that does not trigger ovulation, may also occur in approximately 47% of normally cycling women [141]. These surges may be spontaneous or could be the result of conditions such as polycystic ovary syndrome (PCOS). In addition, luteinized unruptured follicle syndrome (LUFS), a situation in which ovulation does not occur but the unruptured follicle undergoes luteinization and normal production of luteal phase P4 occurs (a subtle cause of infertility [142]) may be present in the athlete population. Importantly, while commercial LH-surge tests give recommendations regarding the day of the menstrual cycle on which to commence testing (based on average cycle length), following these instructions may, on occasion, result in missing the LH surge due to commencing testing too late in the cycle, while some users may cease testing if a positive LH surge is not observed within an expected timeframe. Second, it is important to understand the variation in commercially available LH-surge kits. While digital tools for reading test strips should give consistent readings, visual interpretation of test strips may leave room for error (false positives or negatives). Furthermore, the sensitivity of LH-surge kits may also vary, with ranges from 20 to 50 mLU/mL [143], indicating that athletes and MDTs should be aware of these differences and mindful when switching brands and/or types of LH-surge tests.

Some urinary LH-surge tests can be, or are, combined with other urinary measures to increase the accuracy of LH-surge interpretation. For example, some urinary ovulation kits include a measure of estrogen (E1C). Estrogen has a critical role in the induction of the LH surge [137]; thus, when an increase in E1C is detected, these kits can help predict an impending LH surge. In addition, a metabolite of P4 measured from urine (PdG [144]) may be measured from urine after an LH surge (indicating ovulation) to help assess the quality of the luteal phase. Concentrations of PdG that are ≥ 5 μg/mL over 3 consecutive days, for example, have been shown to confirm ovulation (via ultrasound) [145]; however, thresholds for a positive PdG test will also be kit/manufacturer specific. Taken together, measures of E1C and PdG in combination with the LH surge may be considered in situations where an athlete or MDT seeks information indicating (but not confirming) a eumenorrheic menstrual cycle or menstrual dysfunction, but blood testing is not feasible, available, or desired. Like blood testing, there are several practical limitations to testing urine, including the cost of kits and the additional time burden for the athlete. Furthermore, regular urine testing may not be feasible for all athletes/teams due to financial and logistical constraints, while this kind of testing may become an additional stressor for athletes as well.

With the variety of tools available for tracking menstrual and hormonal contraceptive cycles, it is important to consider the potential for excessive and unnecessary medicalization of menstrual/hormonal contraceptive cycle tracking and female sport, in general. Where tracking menstrual health has several potential benefits for athletes and teams, there are several considerations and risks, which are detailed in Sect. 4 and mentioned throughout the article. Athletes displaying symptoms of any illness or discomfort should be referred to a physician for follow-up and medical support. Comparably, athletes who are healthy/injury free and developing/performing as expected may not benefit from additional tracking.

### Tracking in a Team-Sport Environment and Contextualizing Data

Prior to implementing menstrual health monitoring in a team, there are a number of essential considerations: (1) availability and delivery of education to athletes, coaches, and MDTs, which should include information on why menstrual health data are important to track, what is considered normal/abnormal for cycle characteristics, who to follow-up with should cycle characteristics deviate from “normal,” and how this medical data will be used and protected, including who will have access to it; (2) ensure there is a support system in place for when medical support (MDT or referral to a physician) is required; (3) create a safe space; data should only be shared with individuals to whom consent has been given by each athlete; and provide adequate resources to support best practice for data interpretation and management; and (4) consider cultural and age-specific factors, as there are likely to be differences in attitudes towards menstrual health data. In under-resourced environments and situations where safe and ethical tracking and use of menstrual health data cannot be guaranteed, the focus should be placed on educating and empowering athletes around the topic of menstrual health, including how to track their own cycles and how best to support their own menstrual and overall health, including information about when, and from whom, to seek help.

Once these essentials have been ensured, it may be warranted to recruit personnel that understand both menstrual health and sport-specific football performance to integrate and contextualize these data. Where many teams will struggle with how to collect data, even more teams are likely to struggle with how to organize, analyze, and effectively use and communicate these data to players, coaches, and MDTs. As such, a designated sport scientist or menstrual health advocate within an organization and part of the MDT may be able to ensure that essential practices are in place as suggested in this paper. Within team environments, monitoring menstrual health via the interpretation of multiple data streams can add significant value and context when looking to optimize health and performance. Longitudinal monitoring of athlete health and performance can enable an understanding of the unique “normal” of each athlete, allowing for significant deviations from their “normal” to be identified. In team sports, such as football, individually tracking the hormonal cycles can be a first step in identifying possible cycle-related (phase-based) patterns influencing training and performance [146]. However, if tracking is commenced at a team level, it should be ensured that individual differences in hormonal variation and related personal experiences are considered [147].

The systemic actions of endogenous reproductive hormones E2 and P4 can impact various measures that are commonly tracked in football settings. Where a measure of E2 or P4 that falls within clinical norms will not provide coaches or athletes with pertinent information for planning day-to-day training and recovery, information regarding menstrual status and hormonal contraceptive use as well as menstrual cycle phase (particularly if the athlete repeatedly experiences adverse symptoms) may be valuable to athletes, coaches, or MDTs in order to contextualized other health/performance related data. Athletes need to be able to compete and train on any day of their cycle, so ensuring that they know what is healthy/normal and when to seek help is key.

Performance (and recovery)-related parameters might be affected by the menstrual cycle (menstrual cycle phases, menstrual status, and hormonal contraceptive use) in individual athletes, but the research regarding these effects is comparatively equivocal on a group level [148]. That is, current evidence suggests that strength/endurance performance does not markedly change over the menstrual cycle [149] or hormonal contraceptive cycle [150]; however, the quality of research behind these findings is relatively low [149, 150], thus limiting our ability to draw robust conclusions or provide evidence-based recommendations. Likewise, the use of hormonal contraceptives is suggested to have a small negative influence on skeletal muscle protein turnover and strength development in response to resistance training [151], but hormonal contraceptives do not appear to categorically affect strength or hypertrophy [152]. The variety of hormonal contraceptives available and the lack of controls/detailed reporting regarding hormonal contraceptive use in most of this research also limits our ability to draw robust conclusions or provide evidence-based recommendations. In addition, the influence of menstrual disturbance/dysfunction on performance is not fully elucidated, as current research indicates a decreased potential for positive training adaptations [153, 154], decrease in performance [155], or no effect [156]. Furthermore, although some footballers currently plan their training according to their menstrual cycle [26] and athletes can use information about their menstrual cycle to adapt their training as needed [157], it is worth noting that there is currently no clear scientific evidence to justify the time and resources needed to apply so-called period-periodized training strategies [158, 159] on a team level, although there is also no evidence to prove these strategies are ineffective [159]. These observations indicate that gaps in our understanding of menstrual health on training responses and adaptations exist but also illustrate the potential scientific value of tracking menstrual health in sport context.

The impact of the menstrual and hormonal contraceptive cycles on performance and recovery in elite footballers has not been fully explored. Original research suggests that footballers perceive that their menstrual cycle negatively affects performance [160], but systematic review indicates that controversy exists [161]. Research in other athlete populations suggests that subjective negative effects on performance (and unpleasant symptoms) are similar in both naturally menstruating females and hormonal contraceptive users [23–25, 162], indicating that mental performance (mood, ability to focus) may change over the menstrual or hormonal contraceptive cycle [23, 25]. Perception of the best and worst phases in which to train or perform, in general, seem to be linked to adverse symptomology [23–25], described in Sect. 2.1, while a link between decreased training and competitive performance due to PMS symptoms has also been described in the literature [163]. A higher rating of perceived exertion during football activities (training and matches) has been reported during the luteal phase [164] and after a graded exercise test during menses compared to non-bleeding days [165], although a recent meta-analysis indicates this result is not consistent [166]. Where heart rate variability appears to be influenced by the menstrual cycle [167, 168] and hormonal contraceptive use [169], investigation regarding the effects of the menstrual cycle on measures of sleep quality are not in agreement [170–172], although amenorrhea appears to be associated with poor sleep [173]. In terms of biomarkers that may be regularly used in the field, lactate response to exercise may be affected by menstrual cycle [174, 175], but this observation is also inconsistent [176]. Overall, basic tracking of menstrual cycle characteristics including bleeding days and (pre)menstrual symptoms might already improve health literacy of female athletes while providing valuable information for coaches and MDTs to identify recurring patterns related to adverse symptomology.

This equivocal nature of research results regarding the influence of sex steroids on characteristics of performance, recovery, and health may be due to the variability in approaches to defining and identifying menstrual cycle phase and menstrual status as well as the variety of hormonal contraceptives used, but may also be driven, in part, by the significant intra- and inter-individual nature of menstrual health patterns and subjective experiences. Further high-quality and transparently reported research is needed to elucidate the effects of female sex steroids on performance. Presently, laboratory research appears to focus on eumenorrhea rather than diverse hormone profiles and absolute hormone levels/ratios rather than “hormonal transitions” [177], such as those observed in the premenstrual and menstrual phases, where symptoms are most often reported. As such, diverse hormone profiles that are representative of the athletic and general population deserve research attention, while transitional timepoints should be considered when collecting data regarding menstrual or hormonal contraceptive cycles and performance. Furthermore, there is a paucity of data focusing on elite female athletes, where small changes that may not appear statistically significant can become quite relevant. Overall, however, monitoring menstrual health alongside performance data has the potential to be useful, as characteristics of menstrual health may explain minor but possibly meaningful changes in performance.

## Considerations for Tracking

Although menstrual health tracking may be beneficial for some athletes and teams, we must also be aware of several considerations including potential barriers and limitations related to tracking. While there are several solutions to help navigate the challenges listed herein, it is essential to recognize that collection of any menstrual health data can unintentionally threaten the autonomy, privacy, and safety of female athletes [178, 179] and that many athletes may be uncomfortable discussing their reproductive health [180, 181] as menstrual health-related issues and pregnancy may also threaten their positions on teams and/or contracts/sponsorship [178].

When implementing menstrual health tracking or monitoring of player health and performance, teams/organizations must be aware of ethical guidelines and regulations related to healthcare and research, both of which require voluntary informed consent. Voluntary informed consent must be obtained prior to commencement of data collection, and this consent can be withdrawn, at any time and for any reason, without negative consequences for the player regarding, e.g., playing time, contracts, or MDT/coaching support. According to the Queensland Health Guide to Decision-Making in Health Care (2025), consent can be deemed valid if the player has the capacity to make a decision; consent is given voluntary; the discussion between the player and health practitioner is transparent and involves two-way communication; the information is presented in a language the player understands; the player is advised in simple terms of the risks, benefits, alternative options and details of data collection, and any proposed treatment(s); the information provided, and consent given, is specific to the data collected and healthcare provided; and consent is obtained prior to data collection or procedures, and the player has sufficient time to consider all information prior to giving consent [182]. In addition to voluntary informed consent, research related to data collection must be performed responsibly and, at the very minimum, in adherence with local legislation and guidelines regarding research in humans or clinical research (for example, [183]). Importantly, voluntary participants in research should only be exposed to interventions where the anticipated health or scientific benefits clearly outweigh any potential risks or harm [184].

Monitoring menstrual health must consider data privacy laws in the countries where data are being collected and must be particularly sensitive to personal privacy in countries where information about fertility can potentially be used against an athlete. As with all medical/health data, procedures must be in place to ensure that the data are collected and stored with the consent of athletes, that third parties do not have access to the information, and that the athlete can withdraw their consent to collect/share this information at any time. Athletes, coaches, and MDTs have specific rights and responsibilities that may be regulated by law. For example, the General Data Protection Regulation in the European Union dictates what data can be processed and how it can be used. Data must be processed in a lawful and transparent manner; the specific purposes for which data will be used must be communicated to the individuals from whom data are being collected; data can only be collected for the specific purposes communicated and cannot be used for any other purposes; the data must be kept accurate and up-to-date; the data cannot be stored for longer than necessary or longer than what has been communicated; and the organization(s) storing the data must have technical and organizational safeguards to ensure the data are properly handled and stored [185].

While tracking is reported to increase menstrual health literacy among athletes and their MDT [186], this result is not consistent [187] (for perspectives on improving menstrual health literacy in sport, see [186]). This may suggest that the rationale for tracking is not adequately understood by athletes and/or staff; that tools do not adequately meet the needs of the users; or that the data obtained are not shared in a way that promotes or encourages understanding of menstrual health [188–190]. As the menstrual cycle potentially occurs for at least 40 years of females’ lives, it is easy for constant tracking to become a tedious and bothersome endeavor, while tracking menstrual cycles might cause some athletes to become overly focused on certain parameters and aspects of their menstrual or hormonal contraceptive cycle, leading to anxiety and/or self-limiting beliefs. If athletes or teams do not perceive benefits, this may lead to reduced compliance, which is likely to result in incomplete data and incorrect data interpretation [188]. Hence, the benefits of tracking menstrual and hormonal contraceptive cycles should be clarified from the outset and the burden of tracking should be minimized.

In team environments, negative socio-cultural attitudes towards discussing the menstrual cycle and menstruation with different genders [191–193] need to be recognized along with the feasibility of doing so in different religious contexts [194], in sexual and gender minorities [195], in regions that may not have access to (affordable) internet connectivity and smartphones to use these apps [196], the financial resources required to buy the hormone test kits [197], or the MDT and infrastructure to monitor, interpret, and action the results of the tracking [198].

The rate at which menstrual cycle tracking technologies are developing indicates a global interest in menstrual health but also makes it challenging for MDTs to remain current on available technologies and to evaluate their scientific and clinical validity, reliability, and sensitivity. Regrettably, despite their multitude, studies have demonstrated that many tracking applications are inaccurate or provide conflicting, or superfluous data [190]. Although applications claim to be “evidence based,” they have not necessarily been tested or validated in clinical trials, which influences their functionality and reliability [122, 199, 200]. In addition, even though menstrual cycle dysfunction and hormonal contraceptive use are relatively common in females of reproductive age, most commercially available tracking methods are designed for, and have been evaluated in, females with non-pathological and “textbook” menstrual cycles, limiting their application not only in women with menstrual cycle dysfunction or using hormonal contraceptives but also in those in other life stages such as puberty, postpartum, or perimenopause [190, 195]. Furthermore, as the majority of app developers are men [201, 202], women’s viewpoints and lived experiences may be excluded from the creation of menstrual cycle tracking apps. Importantly, despite ethnic and environmental influences on the menstrual cycle [203, 204], most work to develop and evaluate menstrual cycle tracking technologies contains a relatively high proportion of participants who are educated, employed, white, and largely from high-income countries [205–208]. Regrettably, this limits the generalizability of these tracking technologies in other populations and environments [190]. Appropriate education needs to be put in place in the field, and more emphasis on developing tech to meet real-world needs is required.

Another conundrum to tracking lies in the sheer volume of data collected from users and potentially provided to MDTs. Mobile applications often collect more variables than are strictly necessary or that there is a need for [209], which not only raises questions about how ethical this is but also means that MDTs are tasked with supervising these data for their teams are inundated with individual and team menstrual or hormonal contraceptive cycle data. With a lot of variables and data points, it can be difficult for MDTs to establish which variables are actionable and which to ignore despite having collected them. This is particularly challenging when data may need to be managed and integrated with other health or performance data, further highlighting the need for staff with knowledge of menstrual health and performance.

Another consideration for technology companies owning large amounts of intimate health data is users’ perceptions of the security of their menstrual health data, or lack thereof [192]. This may pose a major barrier to the adoption of tracking technologies for some athletes or organizations. This reticence to use tracking technologies may be informed by data breaches [210]. Indeed, recent research shows that many menstrual cycle tracking apps are vulnerable as they do not have minimum security features, despite hosting sensitive information [211]. Furthermore, 80% of health and medicine-related apps share user data with third parties but are not transparent about this practice with their users [209]. This sharing and commercialization of data, surveillance capitalism, may be legal as users willingly (albeit often unconsciously) provide their data in exchange for app access, but it is ethically murky. Having one’s data shared may be considered an inconvenience or minor embarrassment by some, but in jurisdictions where reproductive rights are policed, these apps may be compelled by the courts of law for these data, which can be used as evidence against the user [212]. There is thus an urgent need to address the security and privacy of these data so that female athletes can approach tracking using these technologies with confidence. In under-resourced environments, basic tracking can be completed by individual athletes using paper (e.g., calendar) and pencil in order to better understand their own bodies, but these data do not necessarily need to be shared unless the athlete has questions, seeks support, or expresses concern. Resources to educate athletes for these purposes should be facilitated, with sensitivity to possible cultural differences, by better resourced environments.

## Summary and Future Research

In football, as in all sports, maintenance of athlete health and well-being is critically important and will support the development of the marginal gains needed to “optimize” sporting performance. Herein, we have attempted to address the key elements of “what can be tracked” and “how it can be tracked” in female athletes/footballers. We have also identified several considerations that should be considered. To summarize, the key take-home points for the athlete, coaching staff, and MDT are:

- Menstrual health tracking can be a valuable part of overall health and performance monitoring in naturally menstruating and eumenorrheic athletes, as well as in those experiencing menstrual disturbance/dysfunction, and may also be useful in athletes using hormonal contraceptives.
- Monitoring menstrual health should be specific to the athlete’s and team’s goals and purposes and should only be implemented with voluntary informed consent from each individual player. Education and rationale should be provided for tracking methods and interpretation of results, while distribution of data should be limited to those included in the informed consent.
- Data collection (e.g., blood samples and transvaginal ultrasound) should only be performed by qualified medical professionals or the players themselves (e.g., LH surge, cervical mucus, symptoms). Tracking hormone concentrations (via blood, saliva, or urine) and use of transvaginal ultrasound is not indicated in junior athletes without medical advice and may only be implemented with voluntary informed consent, which may require parental involvement.
- To date, no single method for tracking menstrual health in the field has been identified as superior to others; i.e., there is no omnibus “gold standard.” There are, however, easily accessible and feasible tracking solutions (pencil and paper, tracking apps, over-the-counter hormone kits) that can be utilized across a broad spectrum of sports settings (professional to recreational). It is critical to:
  - Recognize the strength and limitations of each of the available tools and take into consideration how limitations may affect the gathered data. Educate players that trends are more important than single observations of a prolonged cycle or lack of positive LH surge.
  - Employ and utilize the tools on a regular or systematic basis (i.e., long-term tracking) to ensure practical application of the data and to avoid making conclusions based on individual measurements.
  - Be aware that there are potential cultural, societal, financial, and legal (data privacy) considerations regarding what information can be monitored and who may collect, interpret, and share the data from such tracking tools. Remember that voluntary informed consent means that players can also withdraw consent at any time.
- Teams should consider having dedicated personnel (support staff, sport scientists) to help contextualize menstrual health data for use by coaches and athletes. In under-resourced environments and situations where safe and ethical tracking and use of menstrual health data cannot be guaranteed, the focus should be placed on educating and empowering athletes about menstrual health, including how to track cycles and how best to support menstrual and overall health, including information about when and from whom to seek help.
- Not monitoring anything related to menstrual health and performance can be a missed opportunity to identify patterns/successes/challenges, and to promote and empower female athlete body literacy, health, and well-being.

## Call to Action

As the scientific community continues to advance our knowledge and technology, the goal remains clear: to empower female athletes with the tools they need to perform at their best, while safeguarding their health, well-being, and privacy throughout their menstrual or hormonal contraceptive cycles and in all aspects of their lives. Future research on tracking cycles and monitoring female (athlete) health will continue to be strongly influenced by technological advances in the areas of sports science and medicine. The last few decades have seen an explosion of knowledge and applications in this area. Unfortunately, not all aspects of this progress have been beneficial in the field of tracking (i.e., issues of validity and “developers promising more than they can deliver” remain). Moving forward, researchers and practitioners will need to examine the validity, reliability, and precision of both new tracking methods and tools and work diligently to ensure they do not get the “cart in front of the horse” on what such applications can truly do and promise to the athlete. Furthermore, it is essential that we continue to investigate how menstrual and hormonal contraceptives might affect characteristics of performance and recovery in female athletes.
